# Identification of gene-oriented exon orthology between human and mouse

**DOI:** 10.1186/1471-2164-13-S1-S10

**Published:** 2012-01-17

**Authors:** Gloria C-L Fu, Wen-chang Lin

**Affiliations:** 1 Institute of Biomedical Informatics, National Yang-Ming University, Taipei, Taiwan; 2 Bioinformatics Program, Taiwan International Graduate Program, Institute of Information Science, Academia Sinica, Taipei, Taiwan; 3 Institute of Biomedical Sciences, Academia Sinica, Taipei, Taiwan

## Abstract

**Background:**

Gene orthology has been well studied in the evolutionary area and is thought to be an important implication to functional genome annotations. As the accumulation of transcriptomic data, alternative splicing is taken into account in the assignments of gene orthologs and the orthology is suggested to be further considered at transcript level. Whether gene or transcript orthology, exons are the basic units that represent the whole gene structure; however, there is no any reported study on how to build exon level orthology in a whole genome scale. Therefore, it is essential to establish a gene-oriented exon orthology dataset.

**Results:**

Using a customized pipeline, we first build exon orthologous relationships from assigned gene orthologs pairs in two well-annotated genomes: human and mouse. More than 92% of non-overlapping exons have at least one ortholog between human and mouse and only a small portion of them own more than one ortholog. The exons located in the coding region are more conserved in terms of finding their ortholog counterparts. Within the untranslated region, the 5' UTR seems to have more diversity than the 3' UTR according to exon orthology designations. Interestingly, most exons located in the coding region are also conserved in length but this conservation phenomenon dramatically drops down in untranslated regions. In addition, we allowed multiple assignments in exon orthologs and a subset of exons with possible fusion/split events were defined here after a thorough analysis procedure.

**Conclusions:**

Identification of orthologs at the exon level is essential to provide a detailed way to interrogate gene orthology and splicing analysis. It could be used to extend the genome annotation as well. Besides examining the one-to-one orthologous relationship, we manage the one-to-multi exon pairs to represent complicated exon generation behavior. Our results can be further applied in many research fields studying intron-exon structure and alternative/constitutive exons in functional genomic areas.

## Background

With the increasing availability of genomic and transcriptomic data in numerous species, identifying orthologs and hence extending the functional characterization and gene annotation is of prevalence in comparative and evolutionary genomics. Orthologs are defined generally as genes originated from a common ancestor but are now found in different species after speciation [1]. Previous ortholog identification has focused mainly on designation in gene and protein levels, and novel algorithms and methods were developed for matching human and mouse conserved exons in gene prediction, such as TwinScan [2], DoubleScan [3] and The Conserved Exon Method [4]; many researchers have presented their work in the construction of orthologous gene databases [5-10]. However, with advanced sequencing depth and expansion in transcriptome data, genes are no longer the proper units for interrogation in functional conservation, evolutionary events, and expressional patterns, especially in the field of alternative splicing.

Alternative splicing (AS) is a crucial mechanism in the generation of multiple transcripts in functional diversity from a single gene loci in eukaryotes. It was reported recently that more than 90% of human genes could be alternatively spliced in various tissues and developmental stages [11,12]. Since transcripts that originated from AS often hold high similarity in sequence, it is likely that problematic assignment in gene orthologs would be reported using sequence-based pipelines.

To address this issue, Ho *et al*. utilized the processed transcript units from all alternative and constitutive exons within each transcript region to define the orthologous cluster at the gene level [7]. This strategy yields better comprehensive coverage of gene pairings and more accurate detection for orthologs from in-paralogs. Moreover, Zambelli *et al*. suggest that the orthology relationship should be considered at the transcript level, since transcript isoforms are the individuals subject to protein functions [13]. They proposed a new concept, splicing orthology, to define isoform orthologs from splicing variants sharing similar intron-exon structures in orthologous gene pairs between human and mouse. At the same time, Jia *et al*. argue that AS should be taken into account in the refinement of existing orthologous gene groups at the transcript level, resulting in conceptual groups of orthologous isoforms of functional equivalence [14].

Based on such concepts, it is reasonable to advance the current concept of orthology to the exon level. Genes and transcripts are widely accepted as functional units and materials for orthology studies; however, both are composed of exons. An ensured orthology observed in a gene pair cannot guarantee the orthology of each exon inside. The exon orthology is in higher resolution compared to gene or transcript levels. Establishing the orthologous relationship between exons provides an alternative and detailed way to view the gene orthology, the extension to genome annotation, materials to study intron/exon gain/loss in evolution, and the association with alternative splicing.

However, through the literature survey, no existing report focused on building databases specialized to assignation of orthologous exons in whole genome, except an unpublished web database [15]. This web site defined a large number of unique meta-exons (non-overlapping exons) with orthologs in human, chimpanzee and rhesus macaque with high similarity from the Ensembl database. To avoid ambiguous detection at the expression level, they excluded repetitive exons in any of the three genomes by mapping exons to other species' genomes. Limited to a collection of only unique exons, their work cannot delineate the whole exon orthology in primates, because the orthology relationships in repetitive and duplicated exons were disregarded.

There were also several studies mentioning the organization of orthologous exons, but they were by-products of researches in various fields and just in a partial coverage to genome. Zhang *et al*. generated a dataset of around 99,000 orthologous exon pairs to investigate the divergence between exonic splicing enhancers and silencers after gene duplication from human and mouse. By using both amino acid and nucleotide sequences in the identification of homologous exons, their amount of exon orthologs pairs was limited and only the protein coding exons was taken into consideration [16]. The Alternative Splicing Annotation Project (ASAP) database also created a collection of orthologous exons from 17 multiple alignments of vertebrate genomes and was widely applied and adopted in a few studies [17-19]. This project identified about 80,000~90,000 human and mouse exons having at least one ortholog. Peng *et al*. investigated tandem exon duplications using orthologous exons in ASAP as well [20]. Although these datasets of orthologous exons selected according to their research purpose were smaller and specific, they illustrated the need and importance of constructing an exon orthology database to facilitate the subjects of exon/intron evolution and alternative splicing between species.

Here, we propose a novel gene-oriented exon orthology database to demonstrate genome-wide orthology relationships at the exon level in human and mouse. Because of the ability to distinguish orthologs from in-paralogs and the considerations of alternative splicing, we choose Ho *et al*. gene orthology database, which was developed by our laboratory, as the source data of this work [6,7]. Constraining the exon orthology to gene annotation and structure is more reliable and informative, and could reduce false positive assignments. Deliberating on conservation in exon order and multiple assignments to orthologs, we identify more than 160,000 (92%) non-overlapping exons with orthologs between human and mouse, far beyond the numbers mentioned in previous works. This exon orthology database can enhance the existing gene or splicing orthology annotations, and furthermore, can provide useful information for applications in many fields.

## Results

### Overview of process in orthologous exon database construction

**Figure 1:**
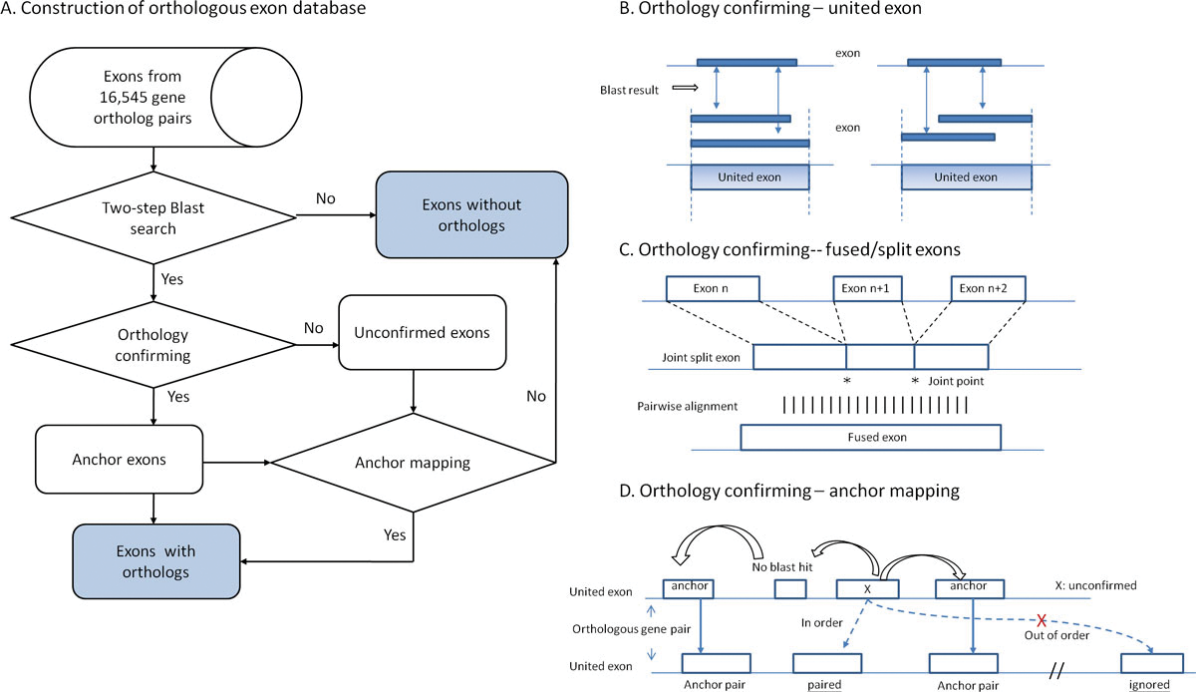
**Process of generating human and mouse exon orthologs.** A. Flow chart of orthologous exon database building. B. Exons with N (N > 1) putative exon orthologs were further verified if these N exons belonged to the same united exon. C. Identification of fused and split exons through analysis procedure from putative orthologous exons. D. Confirmation of the correct orthologous exon pairs via anchor mapping for the rest of the putative orthologs yet to be verified.

From orthologous gene pairs provided from Gene Oriented Ortholog Database (GOOD), our previously developed genome level ortholog database [6], we extract exon information and perform a two-step Blast search (see Method section), which results in the collection of 337,107 putative orthologous exon pairs (Figure 1A) [21]. Due to possible overlapping alternative splicing transcripts, a united exon is defined as the longest part of overlapped exons and is adopted in this study to reduce the complexity of exon relationship assignments. We separate the identification of orthologous exons into two parts: building the anchor exon set and determining the remains by anchor exons. We first select exons that have only one putative orthologs and are best reciprocal hit to each other in pairs and then tag them as anchors. Exons with multiple putative exon orthologs are subsequently checked to determine if they belong to the same united exon. For exons covered by a united exon that shares a certain portion of the sequence, such an assignment would be verified as an orthologous relationship (see Figure 1B). Next, we put fused/split exons into an anchor set from a sub-cluster of exons linked to multiple putative exon orthologs and distinct united exons (Figure 1C).

Taken all together, we obtain a collection of anchors in 325,899 united exons and their orthology relationships are designated. The intron-exon structure is highly conserved between human and mouse was proposed in several previous studies [22,23]; we apply it to generate this comprehensive exon orthology data. Hence, we assume that the proper ortholog of a given exon should be located between the orthologs of the most closed upstream and downstream exon of this given exon, as illustrated in Figure 1D. Consequently, the rest of the putative exon orthologs is determined from the assistance of nearby anchors. One exception is allowed if the putative ortholog with higher conservation both in length and sequence of exon when it is not surrounded by anchors. This special process aims to preserve the multiple orthology relationships derived from duplicate or repetitive exons. Data of exon orthology are available at http://tdl.ibms.sinica.edu.tw/OrthoExon/download.html.

### Orthologs between human and mouse

This study begins with 363,419 exons from human and mouse orthologs and assigns about 92% of exons with orthologous relationships, indicating that most of the exons hold reasonable sequence conservation between human and mouse (Table 1). Related to united exons, the trend remains mostly the same. This investigation shows that human and mouse still have strong conservation at the exon level and supports that they maintain similar gene structures as previously suggested. About 7% of exons possess no orthologous pairs even by a looser criterion, exhibiting the sequence divergence between human and mouse during evolution. As shown in Table 1, only a small portion of exons could not be assigned. Exons/united exons are classified into groups with one single ortholog (one-to-one, 1-1) or with more than one (one-to-multi, 1-N) ortholog. The united exons in the 1-1 subgroup (about 94%) are relatively straightforward; they might evolve within the exons and hold the sequence conservation. However, 1-N cases are complicated. There are many possible scenarios, for example, dividing or duplicating exons [22,24]. In this subgroup, multiple orthologous exons might be derived from the same ancestor region.

**Table 1:** Summary of orthologous exon database

		No orthologs		Orthologs	
			
	All	No hit	**Ignored**^a^** **	1->1	1->N
**Exon**	363419	26312	1501	330407	5199
		7.24%	0.41%	90.92%	1.43%

**United exon**	358067	25149	1200	330541	1177
		7.02%	0.34%	92.31%	0.33%

Here, 1->1 (one-to-one) indicates exons with only one ortholog whereas 1->N (one-to-multi) indicates exons with more than one orthologs. N represents any number that is larger than one. ^a ^putative orthologous exons determined as unreliable after verification and then were ignored in orthologous exon identification.

### Distribution of orthologous exon pairs

**Figure 2:**
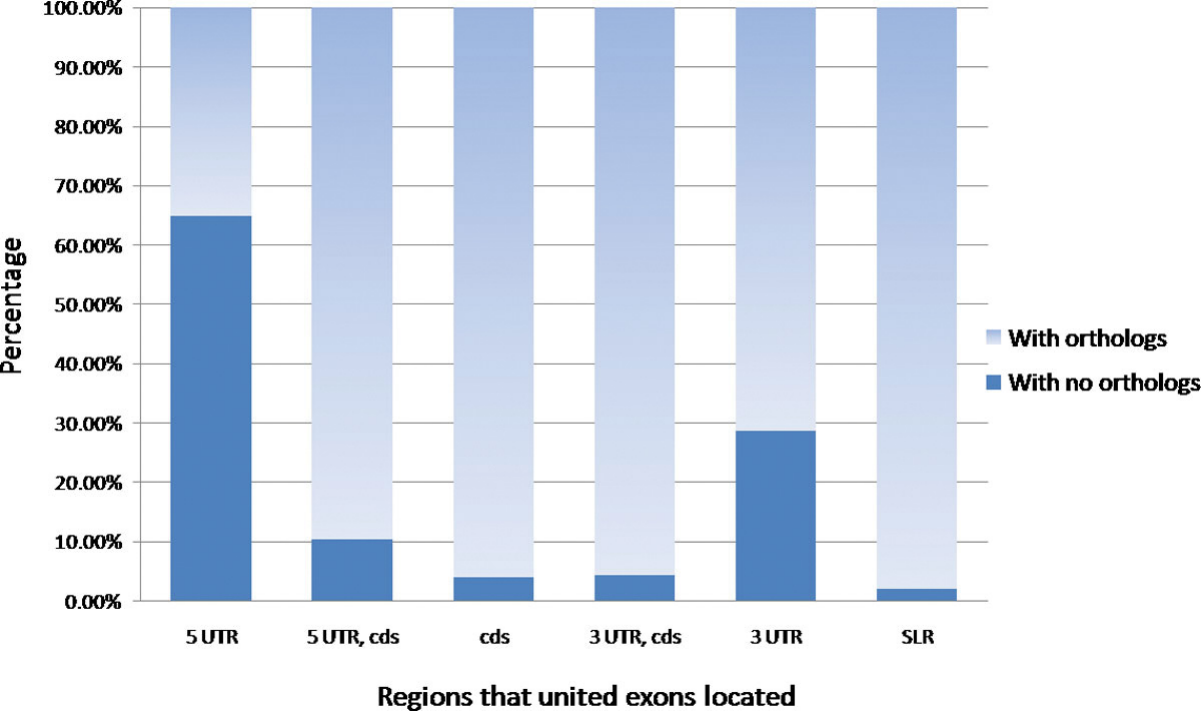
**Distribution of united exons with and without orthologous pairs in different gene regions.** The x-axis represents the gene regions in which united exons are located, and the y-axis is the percentage of united exons with or without orthologs to the total number of united exons in each category. SLR means a single long united exon extending from 5' UTR to 3' UTR.

We further examined the orthologous exon pairs according to the physical regions in which they reside in the gene. Six categories are defined: 5' UTR, 5' UTR with partial coding region, coding region, 3' UTR with partial coding region, 3' UTR, and single long region across 5' UTR, coding region and 3' UTR (SLR). The SLR indicates a united exon that extends from 5' UTR to 3' UTR. In the analysis of distribution in gene regions, the orthologous exons tend to have a higher ratio in coding regions compared to those without orthologs, as expected. In contrast, united exons without orthologs are located more in untranslated regions; for instance, ~39% are in pure 5' UTR, but only 1.67% of united exons having orthologs are in the same place, as shown in Additional file 1. In Figure 2, the united exons located in coding regions can almost find their orthologs (near 95%), implying that the coding region is highly conserved in orthologous exon mapping. Because the RefSeq dataset could not be equal to the completeness of the transcriptome, we assume that the remaining ~5% of the united exons without orthologous pairs might originate from insufficient genome annotation. The birth of new exons or species-specific exons is possible as well [25,26]. In untranslated regions, the percentage of united exons with orthologs dramatically falls to ~71% in 3' UTR and ~35% in 5' UTR, so these regions are not as conserved as coding region is. If we look at two untranslated regions, it is obvious that united exons are more difficult to obtain the orthologous pairs in 5' UTR, ~65% in 5' UTR versus ~29% in 3' UTR, indicating that the 5' UTR region is more diverse at the exon level compared to 3' UTR. Previous studies have reported that recent primate-specific exons tend to reside within UTRs, especially in 5' UTR [26], and 3' UTR could possess regulation binding sites of microRNA in its target gene [27]. These studies suggest 5' UTR might not be as conserved as 3' UTR, which supports our results.

### Orthologous exons in coding region

**Figure 3:**
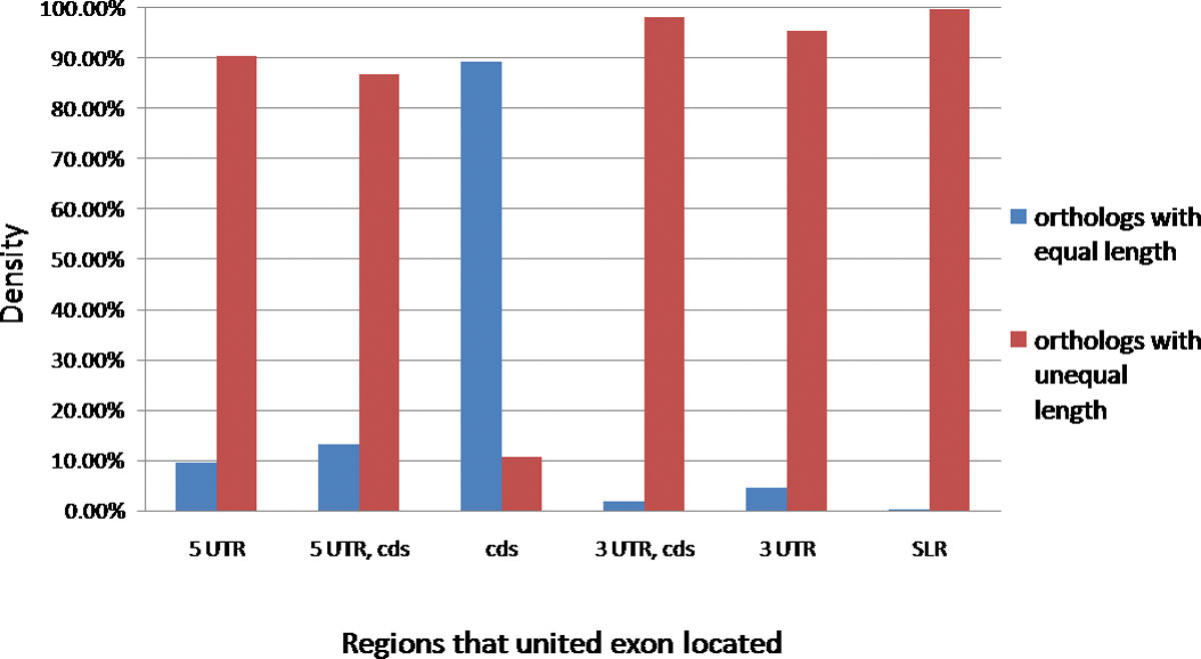
**Comparisons of united exons with or without length conservation to their orthologous pairs from human and mouse.** Only one-to-one orthologous pairs are analyzed in this figure. The x-axis indicates the gene regions in which the united exons are located. The SLR tag means that the united exon crossed through 5' UTR, the coding region, and 3' UTR. The y-axis represents the number of united exons in two categories (with equal or unequal length to orthologous pairs) normalized to the total number of united exons in each gene region.

Exons in an orthologous pair certainly have good similarity in sequence. Our investigation reveals that they have conservation in boundary and nucleotide length as well. In this particular analysis, only united exons with a single ortholog (330,541) are included. About 74% (244,474) of united exons of interest have the same length; many of them fall in the coding region shown in Additional file 2. If we focus on the pure coding region, the ratio of equal length orthologs reaches about 90% of the exon pairs (Figure 3), which means that the united exons in the coding region are constrained not only to sequence but to boundary and length. This reveals that only about 10% of exons in coding region are conserved in sequence homology but not in exon size. Considering to other regions, most of the orthologous pairs (~87% to 98%) differ in length. Interestingly, united exons in 5' UTR have less chance to find orthologs; however, they are more likely to have length conservation compared to those in 3' UTR (Figure 3). United exons in 3' UTR tend to have slightly more diversity in exon length.

### Reading frame correlation between orthologs

**Figure 4:**
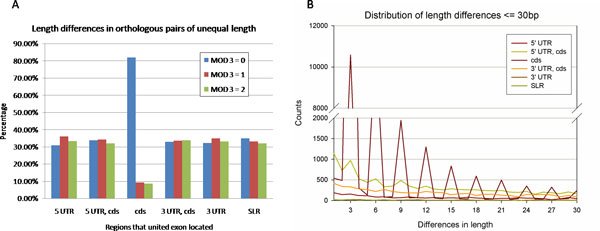
**Analysis of length differences between orthologous pairs of unequal exon length.** A. Distribution of remainders of three in length differences between orthologous exon pairs with unequal length. The MOD 3 = 0 category means that the remainder is zero when the length difference between exon orthologs is divided by three, and so on. The x-axis is the region in which the united exon is located, and the y-axis indicates the percentage of orthologous pairs with unequal length in each region for each category. B. Distribution in counts of united exons where length difference is less than or equal to 30 base pairs compared to orthologous exon pairs.

It is well known that an insertion/deletion whose length is not a multiple of three in protein coding region might trigger a reading frame shift and change the subsequent codons. Regarding united exons without length conservation to their orthologs, we estimate the influences caused by insertion/deletion events. About 26% (86,067) of united exons are of unequal length in orthologs pairs; the differences of lengths are analyzed. Figure 4 shows the distribution of length difference. For such united exons located only in the coding region, the length differences tend to be a multiple of three, which probably keeps the reading frame unaltered. Over 80% (23,388) of united exon pairs of unequal length belong to this type. In contrast to the coding region, the scattering of the three kinds of remainders in the other five gene regions remains at a similar level. The length differences between orthologous exon pairs range from 1 to 21,930 base pairs; about half are less than or equal to 30 bp, suggesting that evolutionary changes in length between exon orthologs are small between mouse and human. Figure 4B shows the length distribution among length difference that are less than 30 bp. Compared to the smoother lines shown in the other untranslated regions, the coding region illustrates several apparent peaks falling in 3, 6, 9 and 12 bp, supporting that length differences tend to be small and be multiples of three. Hiller *et al*. reported that alternative splicing at tandem splicing sites in short distance is widespread in human and mouse genomes [28]; thus we imply that it might be responsible for the subtle changes in exon length here.

### Fused and split exon pairs

Introns hold a larger part of the genome than exons and evolve more quickly. Thus, many events of intron gain and loss must occur during evolution, resulting in a possible exon fusion or fission [29]. If such events occurred in human and mouse, they should be observed in orthologous exon pairs using our dataset. It is reasonable that such cases appeared in united exons with 1-N relationship, as the intron insertion/deletion ends to at least two separated united exons in one genome. Unlike whole gene fusion/fission, exon fusion and split is caused by intron loss/gain, so it might have strict length constraints. Retrogenes make similar phenomena as well [30], even the molecular mechanisms are different. They both contribute to a particular data set that part of consecutive exons in one organism is almost identical to a joint exon in the other one. With a refined discovery pipeline (see Methods section), 64 exon fused/split events were observed and further confirmed, as listed in Additional file 3. Most of them (57 events) correlate to two split exons; this is the most likely scenario, because only one single intron gain/loss event is expected. There are three cases involved in a second intron insertion/deletion event that generate three different exons, as found in Gapdh, Mfap1a, and Mfap1b. Interestingly, two cases, EG433182 and PLEKHA9, possess long fused exons from 11 and 13 split exons, respectively, are reported, and cover almost the entire gene. These two genes are more likely caused by gene duplication induced from copies of retrogenes. To check this assumption, we search the orthologous gene pairs to see if there are more than one ortholog for these two genes. Gene Plekha8 (GOODM_1060172) in mouse is reported to have two orthologs in human: PLEKHA9 (GOODH_0120151), with a long merged exon, and PLEKHA8 (GOODH_1070068), with 13 exons in chromosome 7 [30]. Human gene EG433182 has two orthologs as well: GOODM_1180132, which has a long fused exon, and GOODM_1040527, which has 13 exons in chromosome 4. In biological terms, these two cases are not caused by conventional intron gain/loss events. However, in the view of exon arrangement, they are still assigned to the category of fused/split cases.

## Discussion

With the rapid increase in transcriptome data by expressed tag sequences (EST) and next generation sequencing technology, it becomes important to enlarge the functional annotation to genomes. The assignment of gene functions through orthology is one of the bioinformatic approaches; many efforts have been made to improve accuracy. Recently, transcriptome data have been adopted in the study of orthology to address shortcomings in genome. It is advantageous for its depth and coverage of alternative splicing isoforms. In addition, more and more exon and intron information is being accumulated from transcriptome data, leading the potential to enhance the orthology relationship from the gene/transcript level to the exon level. We utilized the orthologs in GOOD as source materials and propose a new database to identify orthologous relationships in exons between human and mouse. The exons used in our work were derived from reference sequences provided by GOOD; consequently, the comprehensiveness of our database is correlated to the richness of reference sequences and of the GOOD dataset. Since RefSeq database is a subset of transcriptome data in species, the collection of exon information in our study is confined by RefSeq. However, ESTs are thought to contain a large numbers of erroneous and DNA fragments. RefSeq is considered the gold standard for gene and genome annotation.

There are about 7% of non-overlapping exons that can't find orthologs in our algorithm (Table 1); we conclude three reasons responsible for the loss. Firstly, the exon annotation is insufficient. Among the source data of 16,545 orthologous gene pairs, the ratio of alternative spliced genes, which are genes with more than one transcript, is substantially lower than the estimations by other research or databases. Only about 26% and 11% of genes in our dataset have alternative spliced transcripts in human and mouse, respectively, indicating the lack of potential exons supported by various splicing isoforms from other transcriptome data. For instance, human gene CASC4, GOODH_1150074 has two transcripts both containing exon 7, EXH_0175017, in 99 bp, whereas its orthologs in mouse gene GOODM_1020505 also has two transcripts (NM_177054, NM_199038) but lacks an exon of 99 bp in length. Accordingly, EXH_0175017 has no orthologous exons in mouse. Through a manual check in the UCSC genome browser, mouse CASC4 gene has two extra reference sequences, NM_001205369 and NM_001205370, both containing the exon of 99 bp in length. Therefore, the ratio of orthologous exons could be improved in our study by recruiting more transcriptome data and annotations.

The second reason of not finding exon orthologs is originated from sequence divergence in evolution or the limitations in sensitivity and specificity of our algorithm. Human and mouse are organisms between a certain evolutionary distance, and of course some exon sequences have accumulated lots of variations in sequences, especially in untranslated regions. Reasonably, our pipeline might cause false negative detection as well. In observation of real data, we did find out some false negative cases by performing other pairwise alignment programs.

Thirdly, species-specific exons or recently born exons after speciation also lead to the similar phenomena and they are more of biological meanings. The 8^th ^united exon (EXH_1087295) of 138 base pairs, in human nuclear prelamin A recognition factor (NARF, GOODH_1170481) is reported to have no any ortholog in mouse according to our finding, but it is not caused by the shortness of exon information. Sorek *et al*. reported that this exon in human was derived from a newly exonized Alu sequence through RNA editing [25] and as a result there is no way to find a corresponding exon in mouse. Currently, an exon without orthologs is not easy to determine the outcome is derived from incomplete transcriptome data or the new-born exons, so it's not possible to use systematic analysis to select the sub-set of species-specific and newly born exons from our work.

It is not rare to see tandem segmental duplications within genes [31], in addition, exons born from repetitive sequences, like Alu elements, are frequently observed in humans [25,26]. These biological events would lead to multiple assignments of exon orthologs and we can't evade this challenge even it's complicated and increase many efforts in exon orthology construction. Inclusion of exon duplications and repeats could delineate a more complete scene of whole genome. These exons have constraints in sequence length and they could be applied in the judgement of multiple hits in search orthologs. There are 1,177 united exons assigned more than one orthologs; some of them even have more than a dozen. In our data, just about 2% of human and mouse genes contain exon duplications or repeats, which are quite lower than others' work (~8% in human and ~7% in mouse) [20]. Our estimation is based on exon orthology search; however, genes containing exon duplications don't consequently lead to multiple assignments in exon orthologs. For instance, gene MATN3 hold four tandem exon duplication in both human and mouse and these four exons seem to have certain sequence diversity after speciation; as a result, exons in human find their correct corresponding exons in mouse in the exon orthology constructing process. Accordingly, we infer that the orthologs of the 1,177 united exons are partial set of exon duplications in whole genome that possess higher conservation in sequence homology.

Currently, human and mouse hold the most abundant transcriptome data; that is why we chose to build a prototype of exon orthology database based on these two species. The completeness of the transcriptome data affects the applicability of our work; as a result, it is certainly of importance to increase gene and transcript annotation to help the identification of more exons.

## Conclusions

Orthology study should consider the exon level in order to provide more detailed relationships of the species under gene and transcript structure. Starting from a well-processed orthologous gene collection, we present an abundant and informative database that aims to delineate the exon orthology of whole genomes from human and mouse. We identify over 92% of united exons with orthologous pairs; this outcome can be thought as further gene annotation. Our strategy and consequence provide a new way to evaluate orthology at the exon level and have applications in several fields, like exon-intron structure among evolution, difference in alternative and constitutive exons, intron gain/loss between species and newly born/duplicated exons.

## Methods

### Human and mouse gene ortholog pairs

The orthologous gene data were mainly derived from the previously reported Gene Oriented Ortholog Database (GOOD) [6]. There are 16,545 human-mouse orthologous pairs in total based on human NCBI build 36.3 on March 26, 2008 and mouse NCBI build 37.1 on July 5, 2007 [5]. These pairs were generated from 15,923 human and 16,189 mouse genes, meaning that some genes appear in more than one pair due to highly similar paralogs and duplicated gene loci. The one-to-multi orthologous relationships were transformed into many one-to-one relationships in order to decrease comparison complexity. All the annotations of transcripts information were obtained from RefSeq mRNA collection in UCSC website [32,33].

### Putative identification of orthologous exons by Blast search

For each gene, we extracted sequences of every individual exon and transformed them into small Blast databases accordingly. To locate the pairing exon, human exons were used to perform a BlastN search against their mouse orthologs, and vice versa. The total number of BlastN search in human and mouse were 184,580 and 178,839, respectively, equal to the number of exons appeared in 16,545 gene pairs. We selected an expect value of 1e-5 as a threshold to determine a reasonable BlastN hit, resulting in 157,975 human and 156,757 mouse exons having significant hits. The rest of the exons were thought to contain more divergent nucleotide sequences; hence, tBlastX was used for the subsequent search procedure. There were 26,605 human and 22,082 mouse exons run in tBlastX but only 11,276 human and 11,099 mouse exons having significant results under the expect value of 1e-3. The remaining 15,329 human and 10,983 mouse exons did have not any Blast hit using the two-step mapping protocol. In the end, we obtained 337,107 exons (169,251 in human and 167,856 in mouse) that were mappable to their orthologous genes.

### United exons

Here, an exon is defined as a unique pair of genomic coordinates from alignments between all isoforms of one gene and its genomic loci. Thus it is reasonably normal to observe exons sharing one boundary in common or significant portions of a sequence due to alternative splicing or start/stop site selection. For example, gene GOODH_1010092 (PRDM2) has three transcripts, and its exon EXH_0101125 and EXH_0101131 are 20 base pairs differing in the 5' end because of alternative 3' acceptor sites. For these overlapping exons, the complexity of building orthologous relationships between human and mouse exons increases, as it results in one-to-multi connections. Therefore we adopted the longest part of the overlapped exons as united exons to represent the common region between transcripts to simply orthologous exon relations (Figure 1B). After this union reduction process, 358,067 united exons were found in all human and mouse gene pairs, 4,268 of which comprise more than one exons.

### Identification of orthologous exons

After merging BlastN and tBlastX outcomes, we obtained 351,789 putative exon pairings. Since the Blast hit does not indicate the orthologous exon directly, a further verification step is needed. First, we started with exons having only one hit and examined their backward relationship. If two exons in one pair both had only one hit and are Best_Reciprocal_Hits (BRH) to each other, then they were defined as one-to-one (1-1) orthologs. The total number of exons with one Blast hit is 329,591; after examination, 319,046 exons were confirmed in this step.

For 1-N exons, we checked if these hit exons belonged to the same united exon. If their backward search also linked to the original exon, then these exons were designated as orthologs. This step began with 4,125 exons but acquired 10,428 exons as 1-1 united exons, leaving 374 exons as unconfirmed due to backward relationships that did not fit (Figure 1B).

During evolution, one exon may split into more than one exon or exons may fuse into one; such events have stringent constraints in exon length and this characteristic can be used to determine exon fused/split cases [22]. The following criteria were used to find fused/split exon candidates: 1. the difference between the sum of the length in split exons and the length of fused exon is not more than 5%. 2. The putative split exons must be adjacent and can not be inserted into any other exon. 3. The sum of the aligned fraction in split exons against to fused one is set between 0.75 to 1.1 to allow for sequence divergence. After evaluating the Blast results of the exon dataset, 69 potential fused/split cases were gathered, and a verification procedure was conducted. These putative split exons were joined together to form a long sequence, and a pairwise alignment was performed to examine the similarity of the new ligased sequence (shown in Figure 1C). Only the best alignment was needed; the whole alignment must cross the first and last joint point and cover at least 70% of the fused exon. Five cases were filtered under the sequence reconfirmation, and 64 cases were kept contributing 217 reliable exons labelled as fused/split orthologs.

### Assignment of the remaining exons via anchor mapping

In the previous step, we confirmed 329,691 exons (90.72%) with their orthologs in the other genome. These identified exon pairs were used to help assign the rest of the unpaired exons. There are still 7,416 exons (2.04%) unconfirmed in the Blast hits, so we needed more information to assist in ortholog designation. We assumed that the upstream and downstream exon of a given exon should hold similar arrangements in its orthologous gene pair [22,23]. Thus we took advantage of those previous identified orthologous exon pairs and define them as anchors. Eventually, we obtained 325,899 united exon anchors and left 7,019 exons yet to be verified.

For each unconfirmed united exon, its order in the gene was retrieved from the database. The most closed upstream and downstream anchors of the given united exon were searched, and the positions of the corresponding anchor pairs were extracted from the other species. Figure 1D shows an ideal anchor mapping, although it skips a united exon without any Blast hit. One of the Blast hits directed to the right-most united exon was not between the mapped anchors; therefore, this out-order connection was ignored. If the Blast hit of the given united exon was located in the region with which anchor pairs were coupled, this hit was recognized as orthologous, even if there was more than one pairing returned by the program. In real cases, we applied more rules in this algorithm. First, at least two anchors must exist in the gene. Second, one exception is allowed for an out-order Blast result if it has very similar exon lengths (difference <= 6 bp) compared to the query exon and if the Blast e-value drops to less than 1e-10, which is aimed to keep exon duplication cases and avoid false positive detection. Observing the anchor exons from previous identified orthologous exons, 75.47% are of equal exon length; it seems that a Blast hit highly conserved in length is more likely to be a meaningful one. In the end, 310 out-order exceptions are accepted and tagged to distinguish them from those in-order hits.

Overall 5,819 united exons were confirmed with orthologous pairs and 1,200 were abandoned in this step; 777 cases are lack of anchors and 423 ones have no in-order hit. Combining all filtering work, 8,935 Blast results were ignored and not adopted in the identification of orthologous exon pairs.

## Keywords

Exon orthology; alternative splicing; exon duplication; intron-exon structure.

## Competing interests

The authors declare that they have no competing interests.

## Authors' contributions

GCLF constructed the whole dataset, analyzed the data and wrote the manuscript. WCL supervised this study and helped to edit the manuscript.

## Supplementary Material

Additional file 1 **Summary of united exons in different gene regions.** Click here for file

Additional file 2 **Distribution of one-to-one orthologous pairs in different regions.** Click here for file

Additional file 3 Summary of all fused/split exons found in orthologous exon database. Click here for file
