# Supplementary material for: Identification of gene-oriented exon orthology between human and mouse
Source: BMC Genomics. 2012 Jan 17;13(Suppl 1):S10. doi: 10.1186/1471-2164-13-S1-S10 (PMC3303729; doi:10.1186/1471-2164-13-S1-S10)
Supplement: Additional file 1 — Summary of united exons in different gene regions. [file 1471-2164-13-S1-S10-S1.pdf]

Additional file 1. Summary of united exons in different gene regions.

united exons in	5' UTR	5' UTR, cds	cds	3' UTR, cds	3' UTR	across all
with no orthologs	10245	2960	11399	1286	419	40
	38.88%	11.23%	43.26%	4.88%	1.59%	0.15%
with orthologs	5531	25788	269345	28011	1042	2001
	1.67%	7.77%	81.2%	8.44%	0.31%	0.6%
