# Supplementary material for: Identification of gene-oriented exon orthology between human and mouse
Source: BMC Genomics. 2012 Jan 17;13(Suppl 1):S10. doi: 10.1186/1471-2164-13-S1-S10 (PMC3303729; doi:10.1186/1471-2164-13-S1-S10)
Supplement: Additional file 2 — Distribution of one-to-one orthologous pairs in different regions. [file 1471-2164-13-S1-S10-S2.pdf]

Additional file 2. Distribution of 1-1 orthologous pairs in different regions

| united exons | 5' UTR | 5' UTR, cds | cds | 3' UTR, cds | 3' UTR | across all |
|---|---|---|---|---|---|---|
| of equal length in 1-1 orthologs pair | 533 | 3364 | 239996 | 530 | 47 | 4 |
| | 0.16% | 1.02% | 72.61% | 0.16% | 0.01% | 0 |
| of unequal length in 1-1 orthologs pair | 4983 | 22309 | 28519 | 27276 | 992 | 1988 |
| | 1.51% | 6.75% | 8.63% | 8.25% | 0.3% | 0.6% |
