# Supplementary material for: Identification of gene-oriented exon orthology between human and mouse
Source: BMC Genomics. 2012 Jan 17;13(Suppl 1):S10. doi: 10.1186/1471-2164-13-S1-S10 (PMC3303729; doi:10.1186/1471-2164-13-S1-S10)
Supplement: Additional file 3 — Summary of all fused/split exons found in orthologous exon database. [file 1471-2164-13-S1-S10-S3.pdf]

Additional file 3. Summary of all fused/split exons found in orthologous exon database. ('SLR' represents exons that lie across 5' UTR, cds and 3' UTR and column 'intron' is length of intron joining the split exons)

Gene	Exon_id	length	Region	Order	Position	Exon_id	Length	Region	Order	Position	intron
Tnpo1	EXM_0072766	267	cds	17	chr13:99625533-99625267	EXH_0128801	108	cds	17	chr5:72224722-72224829	
						EXH_0128802	159	cds	18	chr5:72224960-72225118	132
Arbp	EXM_0121951	264	cds	3	chr5:116010750-116011013	EXH_0067869	176	cds	4	chr12:119121671-119121496	
						EXH_0067868	88	cds	5	chr12:119121391-119121304	106
KRT8	EXH_0065239	291	cds	5	chr12:51580116-51579826	EXM_0080979	165	cds	5	chr15:101829369-101829205	
						EXM_0080978	126	cds	6	chr15:101828947-101828822	259
LOC644907	EXH_0138111	505	SLR	1	chr7:44473967-44474471	EXM_0032984	46	5 UTR	1	chr6:115758761-115758716	
						EXM_0032983	101	5 UTR, cds	2	chr6:115757920-115757820	797
						EXM_0032982	182	cds	3	chr6:115757185-115757004	636
						EXM_0032981	175	3 UTR, cds	4	chr6:115755706-115755532	1299
Birc6	EXM_0181649	384	cds	7	chr17:74965131-74965514	EXH_0111403	223	cds	7	chr2:32479735-32479957	
						EXH_0111404	161	cds	8	chr2:32480035-32480195	79
Rplp1	EXM_0048777	239	3 UTR, cds	3	chr9:61761328-61761090	EXH_0175944	118	cds	3	chr15:67534563-67534680	
						EXH_0175945	118	3 UTR, cds	4	chr15:67534821-67534938	142

**Ptbp1**	EXM_0152313	320	cds	4	chr10:79321697-79322016	EXH_0188967	173	cds	4	chr19:755036-755208	
						EXH_0188968	147	cds	5	chr19:755292-755438	85
**Rexo1**	EXM_0057478	260	cds	14	chr10:80005569-80005310	EXH_0089153	139	cds	14	chr19:1767568-1767430	
						EXH_0089152	121	cds	15	chr19:1767344-1767224	87
**Tlk1**	EXM_0008910	182	cds	15	chr2:70560145-70559964	EXH_0015223	92	cds	15	chr2:171571819-171571728	
						EXH_0015222	90	cds	16	chr2:171571645-171571556	84
**Serinc1**	EXM_0056167	261	cds	6	chr10:57243022-57242762	EXH_0036119	170	cds	6	chr6:122814901-122814732	
						EXH_0036118	91	cds	7	chr6:122814598-122814508	135
**EG433182**	EXM_0183474	1651	SLR	2	chr18:48206387-48208037	EXH_0000681	94	5 UTR, cds	2	chr1:8857563-8857470	
						EXH_0000680	96	cds	3	chr1:8854632-8854537	2839
						EXH_0000679	59	cds	4	chr1:8853156-8853098	1382
						EXH_0000678	70	cds	5	chr1:8850703-8850634	2396
						EXH_0000677	134	cds	6	chr1:8849896-8849763	739
						EXH_0000676	223	cds	7	chr1:8849147-8848925	617
						EXH_0000675	198	cds	8	chr1:8848128-8847931	798
						EXH_0000674	202	cds	9	chr1:8846738-8846537	1194
						EXH_0000673	109	cds	10	chr1:8845989-8845881	549
						EXH_0000672	59	cds	11	chr1:8845591-8845533	291
						EXH_0000671	426	3 UTR, cds	12	chr1:8844075-8843650	1459
**Ddx18**	EXM_0003133	315	cds	6	chr1:123458172-123457858	EXH_0113785	200	cds	6	chr2:118295908-118296107	

						EXH_0113786	115	cds	7	chr2:118296193-118296307	87
Psmc5	EXM_0160885	288	cds	5	chr11:106122737-106123024	EXH_0186139	57	cds	5	chr17:59261225-59261281	
						EXH_0186140	231	cds	6	chr17:59261363-59261593	83
1110032A13Rik	EXM_0090232	220	cds	2	chr18:80394622-80394403	EXH_0188891	43	cds	2	chr18:75897656-75897698	
						EXH_0188892	177	cds	3	chr18:75898318-75898494	621
Git1	EXM_0158450	384	cds	14	chr11:77318370-77318753	EXH_0083256	153	cds	14	chr17:24927818-24927666	
						EXH_0083255	231	cds	15	chr17:24927594-24927364	73
Eef2	EXM_0152716	253	cds	7	chr10:80642289-80642541	EXH_0089452	114	cds	7	chr19:3932450-3932337	
						EXH_0089451	139	cds	8	chr19:3931977-3931839	361
NARS	EXH_0088574	159	cds	3	chr18:53434205-53434047	EXM_0089891	66	cds	3	chr18:64671703-64671638	
						EXM_0089890	93	cds	4	chr18:64671531-64671439	108
Crip2	EXM_0165260	153	cds	2	chr12:114381670-114381822	EXH_0174060	95	cds	2	chr14:105015648-105015742	
						EXH_0174061	58	cds	3	chr14:105015832-105015889	91
Mfap1b	EXM_0011336	538	cds	2	chr2:121296052-121295515	EXH_0074521	220	cds	2	chr15:41896938-41896719	
						EXH_0074520	130	cds	3	chr15:41894564-41894435	2156
						EXH_0074519	188	cds	4	chr15:41894178-41893991	258
RPS2	EXH_0077412	210	3 UTR, cds	7	chr16:1952272-1952063	EXM_0178421	92	cds	5	chr17:24858568-24858659	
						EXM_0178422	120	3 UTR, cds	6	chr17:24858748-24858867	90
Ap3d1	EXM_0057542	380	cds	13	chr10:80182133-80181754	EXH_0089225	149	cds	15	chr19:2072310-2072162	
						EXH_0089224	231	cds	16	chr19:2072091-2071861	72

GPS1	EXH_0187260	301	cds	6	chr17:77605675-77605975	EXM_0161887	165	cds	4	chr11:120647616-120647780	
						EXM_0161888	136	cds	5	chr11:120647866-120648001	87
LOC100042812	EXM_0045314	419	SLR	1	chr8:109963467-109963049	EXH_0165960	178	cds	3	chr12:54722476-54722653	
						EXH_0165961	131	cds	4	chr12:54723414-54723544	762
						EXH_0165964	102	3 UTR, cds	5	chr12:54724170-54724271	627
						EXH_0165962	103	3 UTR, cds	6	chr12:54724170-54724272	627
2410018C20Rik	EXM_0044323	437	5 UTR, cds	1	chr8:86781225-86780789	EXH_0190631	198	5 UTR, cds	1	chr19:13736337-13736534	
						EXH_0190632	239	cds	2	chr19:13736685-13736923	152
Dync1h1	EXM_0164931	185	cds	67	chr12:111899565-111899749	EXH_0173690	61	cds	67	chr14:101578318-101578378	
						EXH_0173691	124	cds	68	chr14:101578474-101578597	97
Mfap1a	EXM_0011343	538	cds	2	chr2:121328605-121328068	EXH_0074521	220	cds	2	chr15:41896938-41896719	
						EXH_0074520	130	cds	3	chr15:41894564-41894435	2156
						EXH_0074519	188	cds	4	chr15:41894178-41893991	258
Hsp90ab1	EXM_0086554	357	cds	7	chr17:45706775-45706419	EXH_0134360	166	cds	7	chr6:44326763-44326928	
						EXH_0134361	191	cds	8	chr6:44327133-44327323	206
Usf1	EXM_0098688	224	cds	9	chr1:173347772-173347995	EXH_0007401	95	cds	10	chr1:159277084-159276990	
						EXH_0007400	129	cds	11	chr1:159276744-159276616	247
Abcf2	EXM_0024689	355	cds	4	chr5:24079594-24079240	EXH_0041622	183	cds	4	chr7:150552133-150551951	
						EXH_0041621	172	cds	5	chr7:150551868-150551697	84

**Rps18**	EXM_0085735	189	cds	3	chr17:34089536-34089348	EXH_0133337	87	cds	3	chr6:33351552-33351638	
						EXH_0133338	102	cds	4	chr6:33351720-33351821	83
**TTN**	EXH_0015434	5609	cds	311	chr2:179108822-179103214	EXM_0009123	5459	cds	308	chr2:76552269-76546811	
						EXM_0009122	150	cds	309	chr2:76546654-76546505	158
**Amd1**	EXM_0055852	238	cds	6	chr10:40010421-40010184	EXH_0135420	145	cds	7	chr6:111320195-111320339	
						EXH_0135421	93	cds	8	chr6:111320631-111320723	293
**UBE2D2**	EXH_0130449	1676	3 UTR, cds	8	chr5:138986525-138988200	EXM_0183061	138	3 UTR, cds	7	chr18:35965172-35965309	
						EXM_0183062	1489	3 UTR	8	chr18:35965329-35966817	21
**Pabpc4**	EXM_0115588	233	3 UTR, cds	16	chr4:122975104-122975336	EXH_0002806	131	cds	14	chr1:39800462-39800332	
						EXH_0002805	103	3 UTR, cds	15	chr1:39800046-39799944	287
**Rpl18**	EXM_0131492	194	cds	5	chr7:52975664-52975857	EXH_0093543	124	cds	5	chr19:53811271-53811148	
						EXH_0093542	70	cds	6	chr19:53811015-53810946	134
**Rps2**	EXM_0178420	334	cds	4	chr17:24857914-24858247	EXH_0077414	174	cds	5	chr16:1952911-1952738	
						EXH_0077413	160	cds	6	chr16:1952658-1952499	81
**GPS1**	EXH_0187249	313	cds	5	chr17:77605663-77605975	EXM_0161887	165	cds	4	chr11:120647616-120647780	
						EXM_0161888	136	cds	5	chr11:120647866-120648001	87
**Arfgef1**	EXM_0000109	240	cds	11	chr1:10179759-10179520	EXH_0043338	100	cds	11	chr8:68342231-68342132	
						EXH_0043337	140	cds	12	chr8:68342019-68341880	114
**Arvcf**	EXM_0175282	1030	cds	5	chr16:18397390-18398419	EXH_0098309	527	cds	5	chr22:18349260-18348734	

						EXH_0098308	500	cds	6	chr22:18347765-18347266	970
Polr2e	EXM_0057332	197	cds	3	chr10:79500129-79499933	EXH_0089009	116	cds	3	chr19:1042906-1042791	
						EXH_0089008	81	cds	4	chr19:1041987-1041907	805
Osgepl1	EXM_0094787	354	cds	4	chr1:53376787-53377140	EXH_0015984	205	cds	4	chr2:190327316-190327112	
						EXH_0015983	149	cds	5	chr2:190327035-190326887	78
Gapdh	EXM_0033672	298	cds	3	chr6:125113454-125113157	EXH_0164044	100	cds	3	chr12:6515921-6516020	
						EXH_0164045	107	cds	4	chr12:6516111-6516217	92
						EXH_0164046	91	cds	5	chr12:6516347-6516437	131
Rps2	EXM_0178419	198	cds	3	chr17:24857479-24857676	EXH_0077416	90	cds	3	chr16:1954367-1954278	
						EXH_0077415	108	cds	4	chr16:1953258-1953151	1021
Scyl1	EXM_0090967	350	cds	3	chr19:5770552-5770203	EXH_0160645	123	cds	3	chr11:65049968-65050090	
						EXH_0160646	227	cds	4	chr11:65050171-65050397	82
Ppp2r5d	EXM_0086811	169	cds	8	chr17:46823380-46823212	EXH_0134070	60	cds	10	chr6:43083917-43083976	
						EXH_0134071	109	cds	11	chr6:43084083-43084191	108
Ndufs2	EXM_0005050	188	cds	5	chr1:173169028-173168841	EXH_0107146	113	cds	6	chr1:159445897-159446009	
						EXH_0107147	75	cds	7	chr1:159446271-159446345	263
Srm	EXM_0117267	230	cds	5	chr4:147967877-147968106	EXH_0000797	84	cds	5	chr1:11038738-11038655	
						EXH_0000796	146	cds	6	chr1:11038570-11038425	86
Abcf1	EXM_0086223	285	cds	16	chr17:36096665-36096381	EXH_0132809	113	cds	17	chr6:30661635-30661747	
						EXH_0132810	172	cds	18	chr6:30661890-30662061	144
PRDM10	EXH_0062551	315	cds	19	chr11:129290123-129289809	EXM_0141840	144	cds	15	chr9:31166593-31166736	
						EXM_0141841	171	cds	16	chr9:31167334-31167504	599

**Ap1m2**	EXM_0046848	279	cds	4	chr9:21110215-21109937	EXH_0090573	131	cds	4	chr19:10553554-10553424	
						EXH_0090572	148	cds	5	chr19:10553310-10553163	115
**PLEKHA9**	EXH_0064379	1579	SLR	3	chr12:43854692-43853114	EXM_0125681	117	5 UTR, cds	2	chr6:54563086-54563202	
						EXM_0125682	156	cds	3	chr6:54565209-54565364	2008
						EXM_0125683	125	cds	4	chr6:54566627-54566751	1264
						EXM_0125684	159	cds	5	chr6:54569188-54569346	2438
						EXM_0125685	41	cds	6	chr6:54570266-54570306	921
						EXM_0125686	158	cds	7	chr6:54572115-54572272	1810
						EXM_0125687	157	cds	8	chr6:54574069-54574225	1798
						EXM_0125688	86	cds	9	chr6:54574547-54574632	323
						EXM_0125689	59	cds	10	chr6:54578840-54578898	4209
						EXM_0125690	131	cds	11	chr6:54579814-54579944	917
						EXM_0125691	71	cds	12	chr6:54580498-54580568	555
						EXM_0125692	62	cds	13	chr6:54585704-54585765	5137
						EXM_0125693	250	3 UTR, cds	14	chr6:54590778-54591027	5014
**Pa2g4**	EXM_0059330	213	cds	8	chr10:127997350-127997138	EXH_0166001	79	cds	8	chr12:54790450-54790528	
						EXH_0166002	134	cds	9	chr12:54790610-54790743	83
**Nudc**	EXM_0022498	315	cds	5	chr4:133090457-133090143	EXH_0102000	117	cds	5	chr1:27141738-27141854	
						EXH_0102001	195	cds	6	chr1:27141949-27142143	96
**Gapdh**	EXM_0033671	198	cds	4	chr6:125113058-125112861	EXH_0164047	116	cds	6	chr12:6516528-6516643	

						EXH_0164048	82	cds	7	chr12:6516736-6516817	94
GAPDH	EXH_0164049	413	cds	8	chr12:6517011-6517423	EXM_0033670	231	cds	5	chr6:125112726-125112496	
						EXM_0033669	182	cds	6	chr6:125112411-125112230	86
Dnmt1	EXM_0046713	281	cds	37	chr9:20713196-20712916	EXH_0090432	167	cds	37	chr19:10107963-10107797	
						EXH_0090431	117	cds	38	chr19:10107528-10107412	270
Pcx	EXM_0184704	379	cds	13	chr19:4618142-4618520	EXH_0060453	222	cds	16	chr11:66376707-66376486	
						EXH_0060452	157	cds	17	chr11:66375993-66375837	494
Wars	EXM_0069516	287	cds	8	chr12:110104509-110104223	EXH_0073063	113	cds	9	chr14:99879477-99879365	
						EXH_0073062	174	cds	10	chr14:99878661-99878488	705
Jup	EXM_0064923	276	cds	9	chr11:100238312-100238037	EXH_0084303	156	cds	9	chr17:37168648-37168493	
						EXH_0084302	120	cds	10	chr17:37168296-37168177	198
CEP250	EXH_0196113	179	cds	14	chr20:33524613-33524791	EXM_0106207	136	cds	10	chr2:155795731-155795866	
						EXM_0106208	43	cds	11	chr2:155795930-155795972	65
BC006779	EXM_0014099	354	cds	16	chr2:180964614-180964261	EXH_0096980	135	cds	17	chr20:61662726-61662592	
						EXH_0096979	219	cds	18	chr20:61662494-61662276	99
Gnas	EXM_0107234	180	cds	16	chr2:174170884-174171063	EXH_0197144	59	cds	14	chr20:56917971-56918029	
						EXH_0197145	121	cds	15	chr20:56918134-56918254	106
Ncln	EXM_0057726	319	cds	8	chr10:80952865-80952547	EXH_0189397	140	cds	8	chr19:3155003-3155142	
						EXH_0189398	179	cds	9	chr19:3155571-3155749	430
Abcf1	EXM_0086228	187	cds	11	chr17:36098174-36097988	EXH_0132803	101	cds	11	chr6:30659364-30659464	
						EXH_0132804	86	cds	12	chr6:30659561-30659646	98
